# Potential of Industrial Pineapple (*Ananas comosus* (L.) Merrill) By-Products as Aromatic and Antioxidant Sources

**DOI:** 10.3390/antiox10111767

**Published:** 2021-11-04

**Authors:** Arantzazu Valdés García, María Isabel Domingo Martínez, Mercedes Ponce Landete, María Soledad Prats Moya, Ana Beltrán Sanahuja

**Affiliations:** Analytical Chemistry, Nutrition and Food Science Department, University of Alicante, P.O. Box 99, E-03080 Alicante, Spain; arancha.valdes@ua.es (A.V.G.); midm6@gcloud.ua.es (M.I.D.M.); merxiponce7@gmail.com (M.P.L.); maria.prats@ua.es (M.S.P.M.)

**Keywords:** pineapple by-products, antioxidant activity, volatile compounds, total phenolic content, experimental design

## Abstract

Pineapple is meanly commercially processed. However, it is a fruit that generates a high proportion of nonedible wastes, which are rich in antioxidant compounds and have a varied aromatic profile. These characteristics turn these by-products into potential agri-food waste that can be revalued and applied in different fields such as medical, pharmaceutical, or food applications. The aim of the present work was the characterization and extraction of the volatile compounds present in two pineapple by-products (peel and core) and the subsequent evaluation of their antioxidant capacity. For this purpose, the analysis of the aromatic profile of both by-products has been carried out using the headspace solid-phase microextraction technique coupled to gas chromatography with a mass spectrometry detector (HS-SPME-GC-MS). The optimization of the extraction conditions of the volatile compounds has been validated using a Box–Behnken experimental design. In addition, a quantitative analysis was carried out to determine the contents of two important volatiles in pineapple wastes, isopentyl, and ethyl acetate. Moreover, the estimation of the antioxidant capacity of the subproducts extracts was carried out using different methods All the antioxidant assays demonstrated that pineapple subproducts are rich in easily extractable antioxidants with possible applications in the food industry.

## 1. Introduction

According to FAO, it is estimated that the world production of pineapple (*Ananas comosus* (L.) Merrill) can grow 2.3% per year, up to 33 million tons in 2029 with Asian countries and America as the main producers. In recent years the pineapple market has grown significantly, reporting a clear increase in exports from 3 to 4M tons from 2016 to 2019 [1]. This increase could be due to both its high nutritional value and the competitive sales prices to the public [2]. Pineapple is one of the most appreciated tropical fruits in the world due to its nutritional and organoleptic properties. The pineapple cultivar, level of maturity, climatic conditions, and postharvest handling are factors that significantly impact the chemical and biochemical properties of pineapple [3]. On a nutritional level, pineapple contains mainly carbohydrates and water, followed by dietary fiber, sugars, organic acids, vitamins (ascorbic acid, niacin, and thiamine), and minerals (mainly magnesium, manganese, and copper) Moreover, pineapple residues also contain significant quantities of antioxidant compounds which are known to have a beneficial effect on human health when fed. A recent study has proved that the ingestion of pineapple in rats lowered the hypercholesterolemia-induced cardiac lipid peroxidation risk and inflammation [4]. 

On the other hand, volatile compounds present in the pineapple aroma are key substances that determine the final attributes of fresh and processed pineapples [5]. Regarding the volatile profile, previous works reported esters as main components, contributing 50–80% of the total volatile compounds identified and being related to the quality of the flavor of different pineapple parts [6]. Apart from esters, other volatile compounds present in pineapple are terpenes (2–30%), alcohols (16%), acids (7–14%), aldehydes (10–13%), lactones (2–13%), phenolic compounds (6–12%), and ketones (7–10%) [7,8].

This fruit can be consumed fresh, processed into juice, or packaged in different formats such as pineapple in its juice, slices, natural, etc. [5]. In 2016, 1.45 million tons of pineapple were imported into Europe, of which 50% was treated [1]. During the processing steps, around 60 wt.% of by-products (435,000 tons) such as the crown, peeled skin, and core were generated, which represented around EUR 360,000,000 of economic losses. Traditionally, trying to avoid a negative impact of residues on the environment, these pineapple by-products are usually used for animal feed, disposed of as waste in landfills, or burned for energy production [9]. Nonetheless, the mentioned by-products constitute a potential source of high-added valuable substances, such as volatile compounds, antioxidants, organic acids, sugars, bromelain, and phenolic compounds [5]. However, most of the research related to their revalorization has been focused on the reuse of proteolytic enzymes such as bromelain [10], the extraction of pectin from the peel [11], or the use of juice to produce vinegar [12]. One additional promising possibility is the extraction of the antioxidant compounds from the pineapple by-products to use in the pharmaceutical industry as natural sources of bioactive substances. The potential source of antioxidants and alfa-glucosidase inhibitors in pineapple subproducts was recently verified by Azizan and coworkers in ethanolic extracts [13].

To characterize the volatiles, present in pineapple by-products, Headspace Solid Phase Microextraction (SPME) coupled with Gas Chromatography–Mass Spectrometry (GC-MS) is a relatively inexpensive, easy-to-use analytical technique that is widely applied to volatile compound profile analysis. This technique has also been used to characterize the aroma of pineapples combining extraction and preconcentration in a single step [6,7,8]. Some parameters, such as the extraction temperature and time, salinity of the medium, pH, amount of solvent, and the sample amount and/or volume, among others, directly influence the extraction process using HS-SPME [14]. In this sense, previous studies have described the effect of the addition of a saline solution on the efficiency of the extraction of volatile aromatic compounds in pineapple at different concentration levels [15]. Regarding the extraction temperature in pineapple samples, the reported values of this parameter in studies carried out are between 25–40 °C, while the most suitable extraction time is around 40 and 60 min, depending on the study [6,7,8]. However, optimal operating conditions related to the extraction process of volatile compounds can be obtained using more complex experimental designs, such as the Doehlert matrix (DM), the central compound design (CCD), and three-level designs, such as the Box–Behnken (BBD) design. Focusing on the BBD-type experimental design, it has been used in the extraction of volatile compounds present in fruits such as raspberry [16] or pineapple [17], among others. However, no studies have been found in the literature in which this type of design has been used to optimize the extraction process of volatile compounds present in pineapple by-products, as is shown in this work.

Total Phenolic Content (TPC) can be used as a preliminary screening test of the antioxidant potential of a fruit subproduct. The profile of phenolic compounds and their concentration depends on a diversity of factors such as fruit variety, ripeness, agronomical growing conditions, and so on. Additionally, as the phenolic compounds and other antioxidants compounds must be extracted previously to their determination, the recovery from the products will depend on the solubility in the solvent employed [18]. Considering all these variables it is not strange to find a great variability in results in the literature concerning TPC and antioxidant capacity. Nevertheless, this methodology is widely employed as it is based on colorimetric methods such as the Folin–Ciocalteu method that are very common and easy to use to compare sample processes under the same experimental conditions [19].

Related to pineapple samples, extraction procedures have been carried out by using different solvents. The results showed that the polyphenol content of the extracts was higher when carrying out the extraction in methanol, followed using ethyl acetate and the aqueous extract [20,21]. Other studies have analyzed the content of polyphenols present in the total pineapple by-products [22], while, in the specific case of pineapple peel, the value obtained was around 150 mg GAE 100 g^−1^ of by-product in fresh weight [21].

There are a great variety of antioxidant capacity determination methods in food samples such as 2,2-diphenyl-1-picrylhydrazyl (DPPH), the antioxidant capacity to reduce the ferric ion (FRAP), and the method that uses 2,2’-azinobis (3-ethylbenzothiazoline-6-sulfonate) (ABTS). Studies carried out on pineapple by-products have been obtained for each 0.1 mg mL^−1^ of extract analyzed, an inhibition between 20–70% using the DPPH method [21,22]. On the other hand, by using the FRAP methodology, freshly cut pineapple without treatment presented a value of 7.91 mmol kg^−1^ while the samples treated with chitosan and procydianine showed slightly higher FRAP values of 8.49 and 8.97 mmol kg^−1^, respectively, due to the production of antioxidant coatings on the surface of the sample [20,21]. Finally, values between 63.66–64.02 mM TROLOX g^−1^ of fresh weight were obtained after the application of the ABTS method for the analysis of different pineapple samples treated with different ultrasound power [23]. 

Based on this background, the aim of this work is the extraction and characterization of the volatile compounds present in two different pineapple by-products (core and peel) and the subsequent evaluation of their antioxidant capacity. For this, the evaluation of the antioxidant content in the two pineapple wastes has been performed employing different methods, analysis of the total phenolic content (TPC) in addition to analysis of the antioxidant capacity by using the antioxidant capacity to reduce the ferric ion (FRAP), the 2,2-diphenyl-1-picrylhydrazyl (DPPH) procedure and the method that uses 2,2’-azinobis (3-ethylbenzothiazoline-6-sulfonate) (ABTS). Additionally, the volatile profile analysis of both by-products has been carried out through the headspace solid-phase microextraction technique coupled to gas chromatography with a mass spectrometry detector (HS-SPME-GC-MS). The optimization of the extraction conditions of volatile compounds has been validated using a Box–Behnken (BBD) experimental design. In addition, a quantitative analysis has been carried out to determine the content of ethyl acetate and isopentyl acetate, as characteristic compounds of pineapple, in both by-products. 

## 2. Materials and Methods

### 2.1. Reagents

Methanol (HPLC grade), n-hexane (99%, GC grade), sodium carbonate, sodium chloride, glacial acetic acid, ferric chloride, and potassium persulphate of analytical grade were obtained from Panreac (Barcelona, Spain). Gallic acid monohydrate ACS > 99% (CAS 5995-86-8), phenolic reagent of Folin and Ciocalteu, (±)-6-hydroxy-2,5,7,8-tetramethylchromane-2-carboxylic acid approx. 90% (Trolox) (CAS 238813), 2,2-diphenyl-1-picrylhydrazyl (DPPH) (CAS 1898-66-4), 2,4,6-tris(2-pyridyl)-s-triazine ACS > 99% (TPTZ) (CAS 3682-35-7), 2,2-azinobis (3-ethylbenzothiazoline-6-sulfonic acid) diammonium salt ACS > 98% (ABTS) (CAS 30931-67-0), ethyl acetate, isopentyl acetate and 2-methyl-1-pentanol were purchased from Sigma-Aldrich Inc. (St. Louis, MO, USA).

### 2.2. Pineapple Samples

**Figure 1:**
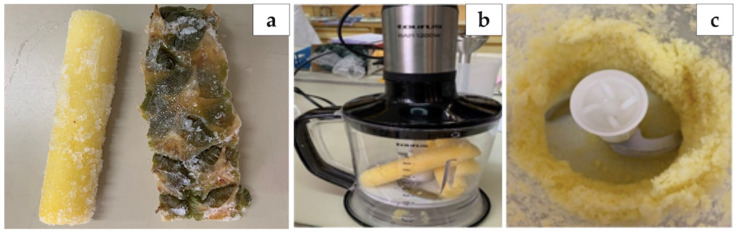
(**a**) Frozen core and peel pineapple by-products used in this study; (**b**) crushing process of the samples with the domestic blender; (**c**) crushed and homogenized pineapple by-products core samples.

Two by-products of pineapple samples, the core (Core S1), and the peel (Peel S1), from Anecoop S. Coop (Murcia, Spain) and two more core (Core S2, Core S3) and peel (Peel S2, Peel S3) samples from two different supermarkets were included in the study (Figure 1a). After receiving the samples, they were immediately vacuum-packed and frozen until their analysis. Before the analysis, samples were firstly crushed with a domestic blender (Figure 1b) for 20 s to homogenize them and reduce their size (Figure 1c).

The crushed samples were used directly for the analysis by HS-SPME-GC-MS. For the study of the antioxidant capacity, it was necessary to extract them in triplicate using a mixture of methanol: deionized water: HCl 1% (70:28:2) according to a methodology previously used by Valdés et al., 2020 [16]. The extracts were stored in the fridge until the analysis was complete.

### 2.3. Antioxidant Capacity Assays In Vitro

Once extracted, samples were subjected to a variety of tests to determine the antioxidant capacity. The methods applied were Folin and Ciocalteu reducing capacity (FRC), the ferric reducing antioxidant power (FRAP), radical scavenging activity by DPPH assay and ABTS cation radical all based on the electron transfer mechanism. The ABTS+ discoloration assay was employed to obtain the Trolox equivalent antioxidant capacity (TEAC). The TEAC assay was previously carried out by Pellegrini et al., 1999 and it was slightly amended for a previous work [24,25]. Then, 200 µL of the extracts were combined with 3 mL of the diluted ABTS solution in a polystyrene disposable cuvette with a lid and then after that it was homogenized in a vortex for 5 s approximately. The reaction mixture was incubated at 25 ± 2 °C for 30 min and afterwards the absorbance was measured at 734 nm. 

The employed FRAP assay was based on the methodology developed previously by Benzie and Strain (1996) [26] but introducing slight modifications that were optimized in a previous work [25]. An extract volume of 200 µL was mixed with 3 mL of FRAP reagent in a polystyrene disposable cuvette and incubated for 40 min sheltered from the light at 25 ± 2 °C. Measurements were conducted with a spectrophotometer at 593 nm.

The DPPH methodology used was adapted from a previous study by Beltran et al., 2019 [25]. An aliquot of 200 µL of the pineapple peel or core extract was added to 2.5 mL of 24 mg a methanol solution of DPPH in a polystyrene cuvette and the kinetics of the reaction was followed monitoring the absorbance spectrophotometrically at 517 nm until the signal reached a stable value. For these samples a stable reaction time of 60 min was selected. Thereafter, all samples were measured in the spectrophotometer at the same wavelength after 60 min of incubation in the dark at ambient temperature. A standard curve was obtained similar to the one obtained in the ABTS, FRAP assays. The results of FRAP, DPPH, and ABTS were expressed as TROLOX micromoles equivalents (TE) per 100 g of pineapple fresh weight (FW).

The FCR assay was carried out based on previous work with some modifications [27]. 300 µL of the methanolic extract was mixed with 100 µL of Folin and Ciocalteu phenol reagent (2 N) and 500 µL of a 7% sodium carbonate solution. The mixture was homogenized with a vortex stirrer for 10 s followed by an incubation period at room temperature for 90 min. Afterward, 5 mL of distilled water was added to each tube and the absorbance of each sample or standard solution was measured at 760 nm in a spectrophotometer using deionized water as blank. Results were expressed as mg gallic acid equivalents (GAE) per 100 g of 100 g of pineapple FW. All antioxidant results obtained were presented in the form of an average of three different extracts for each sample. 

### 2.4. Optimisation of HS-SPME Procedure

To optimize the extraction of volatile compounds from pineapple by-products by the HS-SPME methodology, a Box–Behnken design (BBD) was used with three factors on three levels, and each independent variable was coded between +1, 0, and −1, corresponding to a low, medium, and high level, respectively (Table 1). The experiment selected ranges for the independent variables were extraction temperature (30–70 °C), extraction time (10–60 min), and NaCl concentration (0–4 M). In this work, the proposed BBD consisted of 17 experiments carried out in randomized order, including five central points. The core pineapple sample was selected for the optimization of HS-SPME procedure. The variables were evaluated in a selected ranges on the basis of results obtained in previous analysis and the previous references found [7,8].

The relationship between the response (Y) and the parameters of independent variables was used to monitor the optimization process. The response (Y) was related to the independent variables (X_1_, X_2_, …, X_k_) by using a second-order polynomial model, as can be seen in Equation (1) [28].

Y = β_0_ + Σβ_i_ X_i_ + Σβ_i,i_ X_i_^2^ + ΣΣβ_i,j_ X_i_X_j_ (1)

where Y is the predicted response, X indicates the variables of the process, i and j are design variables, β_0_ is a constant, β_i_ is the linear coefficient, β_ii_ is the quadratic coefficient, and β_ij_ is the interaction coefficient of variables i and j.

The response obtained from the BBD design was evaluated based on the sum of areas obtained for 11 selected volatile compounds: 4 esters (hexanoic acid, methyl ester; 3-(methylthio) propanoic acid methyl ester; 1-butanol-3-methyl acetate; ethyl acetate), 3 terpenes (limonene; α-muurolene; α-copaene), 3 aldehydes (nonanal; decanal; dodecanal) and 1 alcohol (phenylethyl alcohol). All of them have been identified as common and characteristic compounds of the pineapple core in all runs [29].

The satisfactoriness of the fitted model was evaluated by the use of the lack of fit value, the F test, and the coefficient of determination (R_2_) obtained from the analysis of variance [30]. Statistical significance of model parameters was determined at α = 0.05. Additional confirmation experiments (in triplicate) were conducted under the optimal conditions to verify the model validation.

### 2.5. HS-SPME-GC-MS Procedure

Next, 1.0 ± 0.1 g of the sample was weighed into a glass vial (20 mL). Then, 5 µL of internal standard 2-methyl-1-pentanol (560 mg Kg^−1^) was added into the vial, followed by the addition of 2 mL of distilled water (with or without NaCl addition). A polytetrafluoroethylene (PTFE) stirring rod was added to ensure homogeneous stirring of the sample. The fiber used for the extraction of the volatiles was a DVB/CAR/PDMS (divinylbenzene/carboxen/polydimethylsiloxane) 50/30 mm, StableFlex, 1 cm long, mounted to an SPME manual holder assembly (Supelco, Bellefonte, PA, USA) and the procedure was carried out by using the auto-sampler of the equipment. This fiber has been previously used to extract volatile compounds from pineapple samples such as wine [31], juices [32], and pulps from tropical fruits [33].

After the extraction process of volatile compounds, the fiber was immediately desorbed in the spitless mode for 10 min into the injection port of the GC-MS equipment. The equipment used in the analysis was a gas chromatograph (Agilent 7890B) coupled to a quadrupole mass spectrometer (Agilent 5977B). A Gerstel Multipurpose Sampler (MPS) robot was used as the sample introduction system to the chromatograph. The chromatographic column used was an Agilent DB624. The sample was subjected to the following temperature program using helium as carrier gas with a flow rate of 1 mL min^−1^: initial temperature of 40 °C, maintained for 2 min and then an increase in temperature at a rate of 5 °C min^−1^ up to 250 °C, where it was maintained for 10 min. The energy of the ionization source used was 70 eV. The temperature values of the ion source and the transfer line were 230 and 150 °C, respectively. The mass detector was operated in scan mode at a mass-to-charge ratio range (*m*/*z*) of 50–230. In order to verify the absence of contaminants, blank runs were carried out between samples

To identify unknown compounds, the comparison of their mass spectrum data (full scan mode (*m*/*z* 30–550)) with the ones found in the NIST library was carried out. A % of similarity equal or higher than 80% was fixed. In this study, two main pineapple volatile markers were selected: ethyl acetate and isopentyl acetate. Both were quantified in all the studied samples using calibration curves at six concentration levels prepared in deionized water. All determinations were carried out in triplicate.

### 2.6. Statistical Analysis

The study of the fitted model was carried out by using StatGraphics Centurion XV software (Statistical Graphics Corporation, Rockville, MD, USA). SPSS commercial software, ver. 15.0 (Chicago, IL, USA), was used for ANOVA and Tukey’s test at a *p* ≤ 0.05 significance level to obtain differences between values.

## 3. Results and Discussion

### 3.1. Antioxidant Capacity

After applying the FCR, the TPC content in the pineapple core was on average slightly higher than in the peel as shown in Table 2. Core values ranged from 66 to 22 mg GAE 100 g^−1^, presenting all the samples’ significantly different content, meanwhile, in the peel samples the values were comprised between 41 and 15 mg GAE 100 g^−1^. The peel S2 sample was the one with the higher TPC. From the data obtained some variability among samples was found. As was mentioned in the introduction section, the TPC is dependent on the extractant employed in the ripeness stage and the part of the fruit; for that reason it is common to find differences among samples from different origins. Nonetheless, the variability indicated that the values obtained are in the same range of concentration as others found in the literature using similar extractants. Huang et al. [34], for example, found the content of about 75 mg GAE 100 g^−1^ fresh weight in the fruit which is only a little bit higher than the values obtained in the present study in the core and peel of pineapple. In another study, the effect of the extractant was studied over the TPC in three pineapple cultivars. The methanolic extracts evaluated, which were the most similar to the one employed in the present study, showed TPC ranging from 30 to 54 mg GAE 100 g^−1^ of fresh weight [35] and similar variability values among samples to the ones encountered here. 

Regarding the TPC in the pineapple wastes, in a recent study in which different proportions of ethanol in the extractant were assayed, the highest content resulted to be 12.71 mg GAE g^−1^ dry basis of pineapple crown extracts [13]. The peel and core extracts showed slightly lower values, 10.7 and 4.8 mg GAE g^−1^ dry basis, respectively, from 50% ethanol extracts [13]. Considering that the average water content for the core pineapple could be 89.4% [34], the 4.8 mg GAE g^−1^ dry basis found in the core corresponds approximately to 53 mg GAE 100 g^−1^ fresh weight. This value is very close to the one observed in core S1 and core S2 and slightly higher than in core S3. 

Furthermore, other antioxidant assays based on electron transference were applied to compare the antioxidant behavior of the pineapple extracts under a different mechanism of reaction and pH. Thus, the FRAP assay was carried out under acidic pH, the ABTS and DPPH under neutral, and the TPC under basic pH. Independently on the assays employed, some significant differences were observed among the samples of the same type, which corroborates the influence in these parameter factors such as the ripening stage, the cultivar, and growing conditions, among other variables. A comparison with literature is not exactly possible as the values obtained depend on the extractant, the state of the sample, and the extraction conditions. Anyhow, it seems that the values obtained by Huang et al., were in the same range of concentration as the ones obtained here [34]. In that study, different tropical fruits were compared and for the pineapple, they found values near 170 µmol TE 100 g^−1^ in the ABTS and DPPH assay. Nevertheless, the value found by Huang et al. was nearly double the values encountered in the present study. For the three assays, the core and peel antioxidant content were similar; only the peel S2 sample seems to show a higher content compared to the rest. Other data found in the literature are expressed per g of dry extract or sample, and for that reason is not possible to compare the results with those works [9,22,35]. From the data obtained on the core and peel pineapple antioxidants content, it can be remarked that those waste parts have similar antioxidant content as the edible pineapple pulp and that the content is very dependent on the ripeness stage. 

### 3.2. Optimisation of Volatiles Extraction from Pineapple By-Products by HS-SPME 

#### 3.2.1. Model Fitting 

The actual and predicted results of volatile compounds extraction as a response to the different extraction conditions (temperature, time, and NaCl concentration) are tabulated in Table 3. The actual values were like those predicted by the fitted model.

The results of the ANOVA are summarized in Table 4. The determination coefficient (R^2^) of the quadratic regression model was 0.7868, higher than 0.75, which indicates the high suitability of the model. The significance of the model was corroborated by obtaining *p*-value < 0.05. In addition, a significant value of lack of fit (*p* > 0.05) was obtained indicating a reliable model that enough explains the response taking into consideration the variations that can occur within the system. 

According to Table 4, the final second-order polynomial equation is detailed in Equation (2), where A, B, C and D represent extraction temperature, extraction time, and NaCl concentration, respectively:

Sum of areas = 1.37 × 10^9^ − 3.46 × 10^7^ * A + 7.35 × 10^6^ * B − 3.18 × 10^8^ * C + 242177 * A^2^ − 922.35 * A * B + 3.76 × 10^6^ * A * C − 37262 * B^2^ − 909211 * B * C + 2.97 × 10**^7^** * C^2^ (2)

#### 3.2.2. Effect of Independent Variables on Volatiles Extraction 

As can be seen in Table 4, the interaction AC was the most significant factor that affected the extraction of volatiles, followed by the NaCl concentration (C), the quadratic term (C)^2^, extraction time (B) and, finally, the quadratic term A^2^.

Regarding the effect of independent variables, the extraction temperature negatively affected the volatile extraction. This means that when temperature increased, the extraction of target volatile compounds decreased. This fact could be explained because high temperatures may increase the degradation of some of the compounds [36] and the unwanted generation of artifacts [33]. Additionally, a previous study reported similar behavior when HS-SPME was used to extract volatile compounds from tropical fruits, suggesting that low temperature favors the extraction of the more volatile compounds [37]. In this study, this effect was not significant. Regarding the significant and positive effect of the extraction time, in previous works, it has been confirmed that longer times led to a greater retention of the substances until fiber saturation [37]. Regarding the negative and significant effect of NaCl concentration, it has been previously reported that the use of highly concentrated solutions can be responsible for inadequate repeatability values, probably due to adsorption effects at the surface of crystals that can occur in the solution [38]. On the other hand, it is confirmed that the model presents a curvature, due to the significant contribution of the effect of the quadratic terms A^2^ and C^2^.

**Figure 2:**
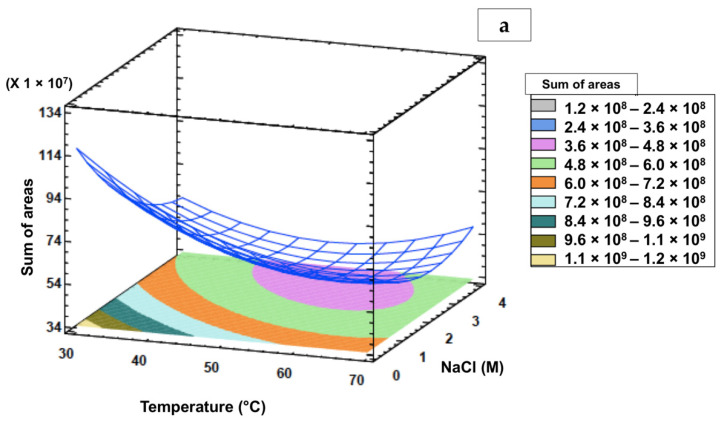

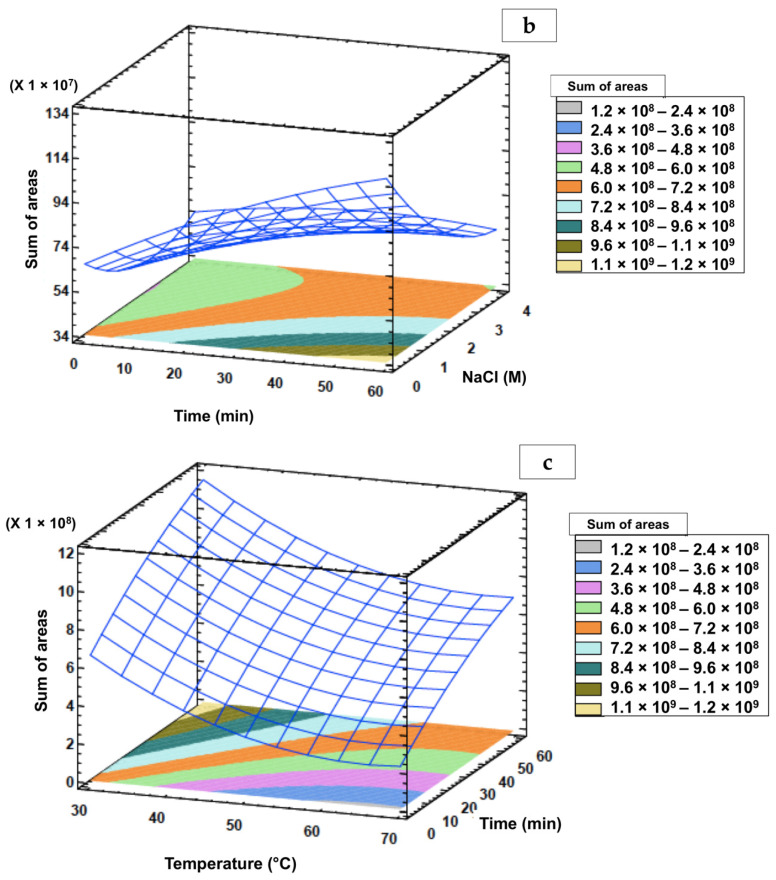
Response surface and contour plots showing interactions on the sum of areas values: (**a**): temperature vs. NaCl with fixed time at 60 min; (**b**) time vs. NaCl with the fixed temperature at 30 °C; (**c**) temperature vs. time with fixed NaCl at 0 M.

After treating the data, response surface figures were obtained to study the interactions between variables to obtain the optimal region and thus verify which independent variable values produced the best response. Three-dimensional contour plots showing the correlation between the factors to the extraction of target volatiles are shown in Figure 2.

Figure 2a illustrates the correlation between temperature and NaCl on the sum of areas of volatiles when the time was kept constant at 60 min. The 3D contour plot portrays an increase in the response when the solution temperature decreases from 60 to 30 °C then a decrease at high temperatures. At low temperature and low NaCl, the response obtained is high. Figure 2b illustrates the correlation between time and NaCl on the sum of areas of volatiles when the temperature was kept constant at 30 °C. The 3D contour plot portrays the increase in response when the solution time increases from 30 to 60 min then the decrease to a lower time. At the high time and low NaCl, the response obtained is improved. Finally, Figure 2c illustrates the correlation between temperature and time on the sum of areas of volatiles when NaCl was kept constant at 0 M. The 3D contour plot portrays the increase in the response when the solution temperature decreases from 50 to 30 °C then the decrease at high temperatures. At low temperature and high time, the response obtained is high. Thus, it could be possible to conclude that NaCl does not enhance the extraction of target volatiles, whereas low temperature and high extraction time benefit the highest response in this study. Similar results have been previously reported for two pineapple varieties (Tainong No. 4 and No. 6) in which 33 characteristic compounds were extracted at 25 °C for 40 min [29] without the addition of NaCl [6]. In a different study, 44 volatiles from the pulp and core from peeled pineapples were extracted at 25 °C for 40 min with low content of NaCl (10:2 ratio of weight sample to NaCl. For minimally processed pineapple fruit, the optimal extraction conditions by HS-SPME obtained through a Doehlert design were 60 min at 35 °C without the addition of NaCl [7]. 

#### 3.2.3. Determination of Optimal Conditions 

The studied response variable was optimized by the combination of the factor levels, which maximizes the response on the indicated region. As a result, the optimal extracting conditions were: 30 °C of temperature, 60 min of extraction time, and NaCl 0.012 M. Under these conditions, the response value predicted by the model was 8.54 × 10^8^. To verify the models, three experiments were performed under optimal conditions with experimental values of 8.09 × 10^8^ ± 1.39 × 10^8^, and did not differ significantly from the predicted value by the model. 

### 3.3. Identification of the Main Volatile Compounds Present in Pineapple By-Products by Using HS-SPME-GC-MS

Based on the results obtained, it can be confirmed that the volatile profile differs significantly when comparing pineapple peel and core samples. The main volatile compounds identified for each type of by-product are shown in Table 5.

In peel samples the main volatile compound found is ethyl acetate, contributing more than 40% to the total area of the main volatile compounds identified, followed by dimethyl sinalediol (6.8–14.7% area); D-limonene (9.6–10.3% area) and isopentyl acetate (3.1–7.8% area). On the other hand, in core samples, octanoic acid ethyl ester is a volatile compound that represents the higher contribution to the total fraction of the main identified compounds accounting for 11.3–16.1% of the total area and followed by 3-methyl-1-butanol (9.0–12.8% area); isopentyl acetate (7.3–12.8% area), ethyl acetate (6.7–12.3% area), and dimethyl silanediol (6.3–9.7% area). These results are following the main volatile compounds found in pineapple samples in previous works [6,8]. 

However, it should be noted that ethyl acetate is considered one of the most characteristic volatile compounds of pineapple because it is responsible for its fruity aroma, as previously described in other studies [7,8,39,40]. This compound is the result of the reaction of acetyl CoA with higher alcohols that are formed from the degradation of amino acids or carbohydrates in pineapple [41]. In addition, the compound isopentyl acetate and other esters such as methyl hexanoate; methyl 3-(methylthio) propanoate, and ethyl 3-(methylthio) propanoate, among others, have also been reported among the prevailing esters in juices made from fresh-cut pineapples. The methyl-branched esters such as isopentyl acetate are well known for their fruity notes and their low odor thresholds in water [42]. In contrast, volatiles other than esters were only present in small amounts in the pineapple juices [43]. Furthermore, the sweet odor of pineapples is attributed to some alcohols such as phenylethyl alcohol and 3-methyl-1-butanol and the group of ethyl esters [41]. 

In the case of the core, the volatiles profile is different as it is shown in Table 5, but the ethyl acetate is also an important volatile. In addition, octanoic acid ethyl ester, 3-methyl-1-butanol, silanediol dimethyl and octane were also important in core samples. It is important to highlight the high contribution of the octanoic acid ethyl ester to the volatile profile of core samples which is related to oily, musty, and rancid flavor but, at low concentrations, it has a nice fruity aroma [31]. 

### 3.4. Quantification of Ethyl Acetate and Isopentyl Acetate Present in Pineapple By-Products by Using HS-SPME-GC-MS

Since the compounds ethyl acetate and isopentyl acetate, among others, are considered key contributors to the pineapple aroma, their quantification using HS-SPME has been carried out in core and peel pineapple samples [6]. The obtained results are shown in Table 6.

In general, peel samples showed higher content in ethyl acetate whereas core samples presented higher contents in isopentyl acetate in comparison with peel pineapple samples. In addition, significant differences are observed among all samples related to the content of ethyl acetate, indicating huge variability that can be related to differences in chemical composition, environmental growth conditions, pineapple cultivar, and maturity stage, among others. 

It has been mentioned previously that the content of ethyl acetate in pineapple samples is the result of the reaction of acetyl CoA with higher alcohols such as isoamyl alcohol that are formed from the degradation of amino acids or carbohydrates [41]. Thus, the composition of the pineapple by-product is directly related to the final content of ethyl acetate quantified. In this sense, the peel is characterized by its high content of cellulose, hemicellulose, lignin, waxes, pectin, and ash. Concerning carbohydrates content, pineapple peel showed higher values in comparison with core samples (17–18 wt.% vs. 13–14 wt.%, respectively) [9,44], this fact being a possible explanation of the higher values of ethyl acetate found in peel samples formed because of the higher amounts of alcohols generated during the degradation of carbohydrates. On the other hand, since isopentyl acetate is one of the prevalent esters found in juices made from fresh-cut pineapples [43], it is expected to be found in fresh pineapple core samples in higher amounts than in the peel fraction of the pineapple as it is shown in Table 6. 

## 4. Conclusions

The resulting TPC values were slightly lower in the peel compared to the core pineapple samples Nonetheless, the antioxidant capacity obtained was fairly similar for both pineapple by-products and there were significant differences among samples. The values obtained in relation to the antioxidant assays confirmed that both pineapple wastes can be a source of antioxidant components, easily extracted and concentrated for different purposes such as an additive in food active packaging or as a natural component of the pharmaceutical industry. 

In this study, the volatile profile of peel and core pineapple samples was compared by employing a Box–Behnken design. The optimal HS-SPME-GC-MS extraction conditions were achieved by using 30 °C of temperature, 60 min of extraction time, and NaCl 0.012 M. It has been corroborated that both parts of the pineapple contain important quantities of volatiles. Therefore, both wastes could be a good source of fruity and sweet aroma useful for their incorporation into food products and active packaging. In this work, the main volatile in pineapple peel resulted to be ethyl acetate. This compound can be used as an additive food enhancer in food and beverages. The same occurs with limonene, isopentyl acetate, and other volatiles encountered in the core of the pineapple. More research work will have to be carried out in the next few years trying to successfully apply pineapple extracts as a source of antioxidants and flavors in the food industry. 

## Figures and Tables

**Table 1:** Coded factors and levels for the BBD.

Independent Variables	Coded Levels		
	−1	0	+1
Temperature (°C)	30	50	70
Time (min)	10	35	60
NaCl (M)	0	2	4

**Table 2:** Total phenol content and DPPH, ABTS, and FRAP antioxidant capacity values obtained for the pineapple core and peel of three samples.

Pineapple Sample	TPC	DPPH	ABTS	FRAP
	mg GAE 100 g^−1^	µmol TE 100 g^−1^	µmol TE 100 g^−1^	µmol TE 100 g^−1^
Core S1	66 ± 5 ^c^	183 ± 10 ^b^	153 ± 3 ^b^	127 ± 11 ^b^
Core S2	54 ± 4 ^b^	156 ± 19 ^a^	126 ± 9 ^a^	131 ± 6 ^b^
Core S3	22 ± 2 ^a^	166 ± 15 ^b^	124 ± 8 ^a^	112 ± 5 ^a^
Peel S1	15 ± 1 ^a^	138 ± 14 ^a^	124 ± 8 ^a^	115 ± 7 ^a^
Peel S2	41 ± 9 ^b^	243 ± 17 ^b^	158 ± 8 ^b^	138 ± 3 ^b^
Peel S3	18 ± 4 ^a^	163 ± 27 ^b^	125 ± 6 ^a^	108 ± 9 ^a^

Values are mean ± standard deviation per 100 g FW (*n* = 3). GAE (gallic acid equivalents) and µmol TE 100 g^−1^ (µmol TROLOX equivalent 100 g^−1^ FW). Values for core or peel samples within the same column with a different superscript letter are significantly different from the others at a *p* < 0.05.

**Table 3:** Combinations of experiment conditions of the BBD and the measured and predicted absolute sum of areas of common volatile compounds in pineapple core.

Run	Temperature (°C)	Time (min)	NaCl (M)	Absolute Sum of Areas	
				Actual	Predicted
1	50	35	2	2.58 × 10^8^	2.52 × 10^8^
2	50	60	4	3.37 × 10^8^	2.87 × 10^8^
3	50	35	2	2.88 × 10^8^	2.52 × 10^8^
4	30	35	4	3.37 × 10^8^	2.88 × 10^8^
5	30	10	2	2.34 × 10^8^	3.11 × 10^8^
6	50	10	0	2.68 × 10^8^	3.17 × 10^8^
7	30	60	2	3.57 × 10^8^	4.56 × 10^8^
8	50	35	2	2.79 × 10^8^	2.52 × 10^8^
9	70	35	0	2.94 × 10^8^	3.47 × 10^8^
10	70	35	4	3.48 × 10^8^	4.74 × 10^8^
11	50	10	4	2.62 × 10^8^	2.34 × 10^8^
12	50	60	0	5.25 × 10^8^	5.52 × 10^8^
13	30	35	0	8.89 × 10^8^	7.63 × 10^8^
14	70	10	2	2.95 × 10^8^	1.97 × 10^8^
15	50	35	2	2.94 × 10^8^	2.52 × 10^8^
16	50	35	2	1.42 × 10^8^	2.52 × 10^8^
17	70	60	2	4.16 × 10^8^	3.40 × 10^8^

**Table 4:** Analysis of variance (ANOVA) for the quadratic model of volatiles extraction.

Source	Sum of Squares		Mean Square	F-Value	*p*-Value
A: Temperature (°C)	2.64 × 10^16^	1	2.64 × 10^16^	6.62	0.0618
B: Time (min)	4.15 × 10^16^	1	4.15 × 10^16^	10.40	0.0321 *
C: NaCl (M)	6.04 × 10^16^	1	6.04 × 10^16^	15.13	0.0177 *
AA	3.95 × 10^16^	1	3.95 × 10^16^	9.89	0.0347 *
AB	8.50 × 10^11^	1	8.50 × 10^11^	0.00	0.9891
AC	9.03 × 10^16^	1	9.03 × 10^16^	22.63	0.0089 **
BB	2.28 × 10^15^	1	2.28 × 10^15^	0.57	0.4916
BC	8.27 × 10^15^	1	8.27 × 10^15^	2.07	0.2236
CC	5.96 × 10^16^	1	5.96 × 10^16^	14.92	0.0181 *
Lack of Fit	7.40 × 10^16^	3	2.47 × 10^16^	6.17	0.0555
Pure Error	1.60 × 10^16^	4	3.99 × 10^15^	-	-
Cor. Total	4.22 × 10^17^	16	-	-	-

* Significant, *p* < 0.05. ** Very significant, *p* < 0.01.

**Table 5:** Volatile compounds identification. retention times, percentage of similarity (%) and % area (vs. total area) obtained by HS-SPME–GC–MS in core and peel pineapple wastes. Nd: not detected.

			Area (%)	
Compound	Retention Time (Min)	Similarity (%)	Pineapple Core	Pineapple Peel
Ethyl acetate	5.65	91	7.1	40.6
Acetic acid	7.60	91	3.9	2.1
1-Butanol, 3-methyl	10.44	91	12.8	2.7
Butanoic acid, 2-methyl, methyl ester	10.99	78	3.9	Nd
Silanediol, dimethyl	11.36	91	9.6	9.3
Octane, 4-methyl	13.21	95	0.2	Nd
Isopentyl acetate	13.60	96	8.3	4.7
Ethylbenzene	14.00	91	1.1	Nd
p-xylene	14.30	97	4.8	Nd
1-Butanol, 3-methyl, acetate	14.68	90	5.1	7.8
1-butanol, 2-methyl acetate	14.78	91	Nd	1.6
Hexanoic acid, methyl ester	16.35	90	Nd	0.9
Hexanoic acid, ethyl ester	18.89	98	6.6	Nd
D-limonene	19.58	98	0.9	9.6
1-octanol	22.03	91	Nd	1.1
Heptanoic acid, ethyl ester	22.16	83	0.5	Nd
3-(Methylthio)propyl acetate	23.92	89	Nd	1.6
Phenylethyl alcohol	24.41	94	4.9	Nd
Octanoic acid, ethyl ester	25.24	87	14.1	Nd
2-Chloro-4-(4-methoxyphenyl)-6-(4-nitrophenyl) pyrimidine	28.01	91	Nd	3.5
Nonanoic acid, ethyl ester	28.12	95	1.7	Nd
alpha cubebene	28.51	90	Nd	2.8
Decanoic acid, methyl	28.96	98	1.7	Nd
alfa copaene	30.42	99	5.6	2.3
n-Decanoic acid	31.49	98	2.6	Nd
alfa muurolene	33.67	99	1.7	0.8

**Table 6:** Volatile concentration (µg Kg^−1^) of ethyl acetate and isopentyl acetate in pineapple core and peel samples.

Samples	Ethyl Acetate	Isopentyl Acetate
Core S1	2406 ± 94 ^b^	483 ± 50 ^b^
Core S2	5103 ± 171 ^c^	476 ± 50 ^b^
Core S3	719 ± 72 ^a^	394 ± 40 ^b^
Peel S1	16540 ± 80 ^f^	88 ± 12 ^a^
Peel S2	13125 ± 75 ^e^	107 ± 3 ^a^
Peel S3	6299 ± 82 ^d^	92 ± 9 ^a^

Values (*n* = 3 ± SD) followed by the same letter, within the same volatile compound, were not significantly different (*p* < 0.05), according to Tukey’s least significant difference test.

## Data Availability

Data are contained within the article.
